# Early intervention in psychosis: An innovation trigger in a challenging environment

**DOI:** 10.1192/j.eurpsy.2021.67

**Published:** 2021-08-13

**Authors:** L. Sile, E. Rancans

**Affiliations:** 1 Doctoral Studies, Rigas Stradins University, Riga, Latvia; 2 Department Of Psychiatry And Narcology, Riga Stradins University, Riga, Latvia

**Keywords:** early intervention, Health care systems, psychosis, schizophrénia

## Abstract

In theory and practice early intervention has an indisputable role in the treatment of patients with psychotic disorders, however modern psychiatrists face challenges in their clinical work to find the balance between the best practice and available treatment options in their environment. One of the biggest challenges remains the implementation of high-cost treatment approaches in healthcare systems of middle-income countries. Moreover, one very important aspect to consider when implementing early intervention is to prepare your team for resistance against the innovation. In countries where psychiatric treatment is more hospital-oriented and out-patient care is limited to short face-to-face visits with psychiatrists, the system is rigid and unwilling to step up to more expanded treatment option, such as a team approach of more than 6 members to treat one patient. In Latvia we started our early intervention programme in 2019 (LAT-EIP), 34 patients enrolled, and 27 finished the programme. When we compared results between standard treatment and LAT-EIP, psychiatrist out-patient visits were comparable to LAT-EIP, but the rate of rehospitalization and assigned disabilities at 12 months follow-up differ dramatically: in LAT-EIP 7.4% had been readmitted and 7.4% were assigned with disability vs 36.1% and 34.4% of patients in standard treatment group, respectively, p<0.05. Nevertheless, the only hospital which continues to provide early intervention is the one which first established it. This presentation will try to explain step-by-step what enhances and what holds back innovation in psychiatry in one middle-income countries.

**Disclosure:**

No significant relationships.

